# Supplementary material for: Information given by websites selling home self-sampling COVID-19 tests: an analysis of accuracy and completeness
Source: BMJ Open. 2020 Nov 6;10(11):e042453. doi: 10.1136/bmjopen-2020-042453 (PMC7650079; doi:10.1136/bmjopen-2020-042453)
Supplement: Supplementary data [file bmjopen-2020-042453supp001.pdf]

Supplementary Table 1: Characteristics of included websites and COVID-19 tests

Name of company	Type of company	Type of test	Name of test manufacturer	Home testing or home sampling	Regulatory approval CE/EUA or *endorsement
Superdrug[40]	Pharmacy	Antibody	Not Reported	Home sampling	-
CityDoc[14]	Private health clinic	Antibody	Not Reported	Home sampling	-
		Molecular	Not Reported	Home sampling	-
Rightangled Healthcare[22]	Online testing specialist	Molecular	Primerdesign Ltd	Home sampling	-
Randox[23]	Laboratory	Molecular	Randox PCR test	Home sampling	-
Samedaydoctor[24]	Private health clinic	Molecular	Not Reported	Home sampling	-
Zava[25]	Private health clinic	Antibody	Not Reported	Home sampling	CE
		Molecular	Not Reported	Home sampling	CE
The Online Clinic (Online Clinic (UK) Limited)[26]	Private health clinic	Antibody	Abbott Laboratories and Roche	Home sampling	-
		Molecular	Not Reported	Home sampling	CE
Doctorcall of Harley Street[15]	Private health clinic	Antibody	Not Reported	Home sampling	CE<br>Endorse UK Government
		Molecular	Not Reported	Home sampling	CE
The Family Planning Association (FPA)[27]	Online sexual health specialist	Molecular	Not Reported	Home sampling	-
YourHealthFirst Clinic: Harley Street[38]	Private health clinic	Antibody	Not Reported	Home testing	CE
Antibody Solutions[28]	Company specialising in beauty treatments	Antibody	Not Reported	Home sampling	-
		Molecular	Not Reported	Home sampling	Endorse PHE, NHS
MyHealthcare Clinic[29]	Private health clinic	Antibody	Abbott	Home sampling	Endorse PHE
		Molecular	Not Reported	Home sampling	-
MedExpress[30]	Pharmacy	Molecular	Not Reported	Home sampling	-
Summerfield Healthcare[16]	Private health clinic	Antibody	Epitope Diagnostics inc (EDI)	Home sampling	-
		Molecular	Not Reported	Home sampling	-
Atruchecks Limited[31]	Online test supplier	Antibody	Abbott	Home sampling	Endorse PHE
		Molecular	Not Reported	Home sampling	-
PillDoctor[32]	Pharmacy	Antibody	Not Reported	Home sampling	-
	Pharmacy	Molecular	Not Reported	Home sampling	Endorse UK Government
London Medical Laboratory[33]	Laboratory	Antibody	Abbott	Home sampling	CE<br>Endorse PHE
		Molecular	Not Reported	Home sampling	CE<br>Endorse PHE
WebMed Pharmacy[34]	Pharmacy	Antibody	Not Reported	Home sampling	CE<br>Endorse PHE
		Molecular	Not Reported	Home sampling	Endorse PHE

Name of company	Type of company	Type of test	Name of test manufacturer	Home testing or home sampling	Regulatory approval CE/EUA or *endorsement
Babylon[39]	Private health clinic	Antibody	Not Reported	Home sampling	-
Medichecks[37]	Online test specialist	Antibody	Not Reported	Home sampling	CE
LondonDoctorsClinic[17]	Private health clinic	Molecular	Not Reported	Home sampling	Endorse PHE, NHS, UK Government
Blue Horizon Medicals[18]	Online test specialists	Antibody	Not Reported	Home sampling	Endorse PHE
		Molecular	Not Reported	Home sampling	-
better2know[19]	Online sexual health specialist	Antibody	Not Reported	Home sampling	CE
		Molecular	Not Reported	Home sampling	-
Private Harley Street Clinic[20]	Private health clinic	Molecular	Not Reported	Home sampling	Endorse PHE
Qured[21]	Private health clinic	Antibody	Not Reported	Home sampling	CE<br>Endorse PHE
		Molecular	Not Reported	Home sampling	CE
LabCorp[35]	Laboratory	Molecular	LabCorp (139900)	Home sampling	EUA
Hims Inc[36]	Private health clinic	Molecular	Rutgers Clinical Genomics Laboratory	Home sampling	EUA

* Statement that the test is 'used by', 'approved by' or 'compliant with'.

Abbreviations: PHE, Public Health England; EUA, Emergency Use Authorisation

Supplementary Figure 1: Reported test manufacturer for the 41 tests for sale included in the cohort.

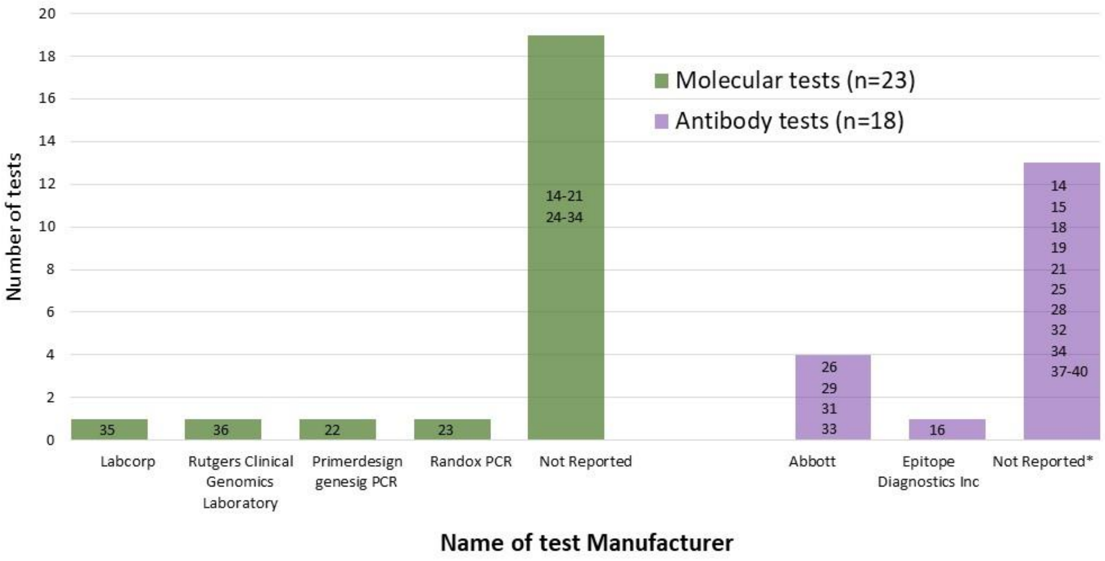

*7 of 13 'Not reported' antibody tests stated it was a finger prick test with no further detail.
